# Mind the gap: Identifying training needs of community health workers to address mental health in U.S. Latino communities during and beyond the COVID-19 pandemic

**DOI:** 10.3389/fpubh.2022.928575

**Published:** 2022-09-12

**Authors:** Luz M. Garcini, Kathryn E. Kanzler, Ryan Daly, Cristina Abraham, Ludivina Hernandez, Raquel Romero, Jason Rosenfeld

**Affiliations:** ^1^Center for Research to Advance Community Health (ReACH), University of Texas Health Science Center at San Antonio, San Antonio, TX, United States; ^2^Department of Medicine, Long School of Medicine, University of Texas Health Science Center at San Antonio, San Antonio, TX, United States; ^3^Department of Psychiatry and Behavioral Sciences, Long School of Medicine, University of Texas Health Science Center at San Antonio, San Antonio, TX, United States; ^4^Department of Family and Community Medicine, Long School of Medicine, University of Texas Health Science Center at San Antonio, San Antonio, TX, United States; ^5^Department of Medicine, University of the Incarnate Word School of Osteopathic Medicine, San Antonio, TX, United States; ^6^Department of Social Sciences, University of Texas at San Antonio, San Antonio, TX, United States; ^7^Center for Medical Humanities and Ethics, Long School of Medicine, University of Texas Health Science Center at San Antonio, San Antonio, TX, United States

**Keywords:** mental health, community health workers, COVID-19, training, underserved and unserved populations, Latino, stress

## Abstract

Addressing mental health is an important part of the COVID-19 response among historically underserved communities, which have been disproportionately affected. Community Health Workers (CHWs) are well placed to offer insights about barriers to mental health service use in their communities, and they are well positioned to address mental health gaps by providing education, resources, and assistance to bridging the gap for the use of more traditional mental health services. Using the perspectives of CHWs, this project identified barriers faced by CHWs in assisting community members with their mental health needs, along with relevant training needs to more effectively deliver mental health resources, referrals, and recommendations to community members. Survey data along with data from focus groups were collected among 43 CHWs in communities that have been historically underserved near the U.S.-Mexico border region. Quantitative data were analyzed using descriptive statistics whereas qualitative data were analyzed through systematic methods. Identified barriers to assisting community members with their mental health needs exist at the personal, community, environmental and organizational levels, and ranged from fear and mistrust to limited services, resources, funding and training opportunities. To help address the aforementioned barriers and facilitate access to mental health service use in their communities, CHWs identified and described opportunities for training in core areas including communication, mental illness symptom identification, trauma, self-care and stress reduction, and cultural awareness and sensitivity. Needs-based training programs that incorporate the insights of CHWs are a crucial part of promoting community-based mental health to address existing mental health disparities in access to and use of mental health services.

## Impact statement

Needs-based training programs that incorporate the insights of Community Health Workers (CHWs) are a crucial part of promoting community-based mental health to address existing disparities in access to and use of mental health services during the COVID-19 pandemic and beyond. Addressing mental health is an important part of the COVID-19 response and in this paper, data gathered from CHWs offer insights about barriers faced and training needs required to properly assist the mental health needs of historically underserved communities.

## What is known about this topic

- Addressing mental health is an important part of the COVID-19 response.
- The COVID-19 pandemic has disproportionately affected mental health in historically underserved communities.
- Historically underserved communities have limited access to mental health services.

## What this paper adds

- Community health workers (CHWs) face multiple barriers to address mental health in their communities.
- Mental health stigma, inadequate funding and limited training opportunities are salient barriers faced by CHW.
- Needs-based training programs for CHWs can help fill gaps in mental health service use in historically underserved communities.

## Introduction

Mental illnesses are on the rise, with ~51 million or 20% of all adults in the United States (U.S.) reporting at least one mental illness in 2019 (1). In the face of the 2019 Coronavirus (COVID-19) pandemic, the high rates of COVID-19 related deaths, compounded economic losses, and the many psychosocial stressors faced by Latinos families have widened existing mental health gaps for the Latino community (2). In 2019, the prevalence of depression symptoms among U.S. Latino adults was 40.3% when compared to 25.3% among their non-Latino White counterparts (3). Although suicide rates declined in 2019 and 2020 when compared to 2018 for most populations, suicide rates among Latino men and non-Latino multiracial women increased in 2020 (4). Disparities in substance use are also evident with 36.9% of U.S. Latino adults reporting an increase or newly initiated substance use when compared to estimates that range between14.3% and 15.6% for other U.S. ethnic/racial groups (3). Addressing long standing mental health disparities among Latino communities in the U.S. is needed to reduce health risk and prevent further harm.

Mental health is often dependent on a variety of factors ranging from genetics to the social and cultural environment, including limited healthcare access. Socioeconomic status, restricted access to opportunities and services, scarcity of resources, limited information, chronic stress, racism, discrimination, marginalization, exposure to hazardous conditions, and inadequate support are often at the root of diminished mental health outcomes as these factors limit the ability of individuals to access needed resources and obtain timely services (5). During the COVID-19 pandemic, underserved Latino communities have faced compounded challenges as a result of increased health risk, widespread mistrust, reduced access to healthcare, barriers imposed by reliance on digital technologies, and limited access to resources that have increase risk of mental illness and psychological distress (6). Undeniably, the COVID-19 pandemic is highlighting deeply entrenched socio-economic and health inequities exacerbating limitations in access to, and quality of healthcare, including mental health services and resources (7).

Mental illnesses can have devastating consequences on the wellbeing of individuals and communities, particularly among people facing social disadvantage. As a leading contributor of disability, mental illnesses are a significant component of the global disease burden. Nonetheless, significant gaps in access to mental healthcare remain despite the existence of low-cost treatment options and effective community-based healthcare approaches (8). Left undiagnosed and untreated, mental illnesses are often comorbid with other chronic health conditions and can negatively impact wellbeing (6). As underserved Latino communities grapple with rising instances of mental illnesses and psychological distress, it is essential to refocus and redistribute resources in ways that reflect the health burden imposed by mental illnesses, particularly stress-related disorders (9).

In the U.S., although mental illnesses are prevalent, the healthcare workforce is often unable to adequately reach historically underserved Latino communities. To address the disparity in access to mental health treatment, the World Health Organization (WHO) proposes collaborating with Community Health Workers (CHWs) to bridge gaps in the provision of health services to communities that have been historically excluded from most healthcare avenues (10). CHWs are frontline public health workers who are trusted members of and/or have an unusually close understanding of the communities they served (11). CHW are also referred to as *promotor/as* in Latino communities; however, for parsimony, we used the term CHW to also refer to *promotor/as*. The wide array of services offered by CHWs include interpretation and translating, providing culturally appropriate resources and health education, counseling and guidance on health issues, serving as advocates for individual and community needs, and in some cases providing first aid and assisting with emergency planning (12, 13). Although most health interventions with CHW have focused on addressing physical health conditions (e.g., diabetes and hypertension) and lifestyle medicine interventions for health behavior change (e.g., nutrition and physical activity), collaboration with CHWs to address mental health is growing (12, 13). For instance, CHWs have been involved in the delivery of mental health interventions to address mental disorders including depression, anxiety, trauma-related distress and other behavioral disorders (13). Results from two recent systematic reviews evaluating the effectiveness of mental health interventions with CHWs show that even though additional empirical evidence is needed, these intervention are promising (12, 13). Indeed, through effective mental health training, CHWs have the potential to address mental health gaps in historically underserved communities, while bridging the gap for the use of more traditional healthcare (14). As trusted members of the communities, CHWs are essential advocates who can speak on behalf of their communities, offer accurate needs assessments, and identify training gaps that may act as barriers to healthcare (15, 16).

Psychological distress arising from exhaustion, poverty, uncertainty, instability, isolation, interpersonal conflict, mistrust, and fear have become part of the daily landscape of care that CHWs have encountered in underserved Latino communities during the COVID-19 pandemic. As community members grapple with the devastating consequences of the pandemic, CHWs can offer guidance, comfort, support, education, and resources to their clients (7). When working with historically underserved communities, CHWs have been effective in addressing the mental health concerns of their clients (16, 17). In fact, CHWs can have a great impact on the wellbeing of individuals who are disenfranchised and least likely to have access to mental health resources (12). As CHWs work through the new challenges imposed by the COVID-19 pandemic, the need for mental health training, adequate personal and professional support, funding and resources is more important than ever (18).

### Purpose

This project was guided through engagement and collaboration with CHWs serving the health needs of historically underserved communities near the U.S.-Mexico border region. Using feedback from collaborating CHWs about the need to address mental health and stress from the COVID-19 pandemic in their communities, the purpose of this project was to identify barriers faced by CHWs in assisting community members with their mental health needs, along with identifying relevant training needs to more effectively deliver mental health resources, referrals, and recommendations to community members during and beyond the COVID-19 pandemic.

### Methods

From its inception, the development of this project relied on shared cultural and contextual knowledge from collaborating CHW organizations, including two CHWs that were included as essential members of the research team. The collaborative process with local and regional CHW organizations began 3 months prior to the launching of this project; this time was used by the principal investigator to identify and learn about the priorities and needs of CHWs and their communities in the targeted region. In the process, the principal investigator along with collaborating CHWs visited community organizations and provided online psychoeducational webinars on self-care for local/regional CHWs. This provided an opportunity for CHWs in the region to become familiar with our work, while our team listened to their needs.

### Data collection

To facilitate obtaining diverse data, purposeful sampling was used in recruitment. CHWs were recruited using networks-based referrals from collaborating CHW organizations across the targeted region (16). Strategies used in recruitment were primarily active approaches including emails, social networks, and presentations offered at local CHW associations. To be eligible to participate, CHWs had to be fluent in English and/or Spanish, and reside and work with historically underserved communities residing in South Texas. No sex or age exclusions were made.

Information was collected using quantitative and qualitative data to obtain in-depth information about novel situations from the COVID-19 pandemic in the targeted communities. Quantitative data was collected using an online survey *via* Qualtrics, whereas qualitative data was collected using online focus groups to comply with health recommendations to prevent the spread of COVID-19. All participants completed the survey prior to participating in a focus group to expand on the content of the surveys. The survey was administered in English or Spanish to accommodate to the preferences of the participants, and it took ~45 min to complete. Surveys and focus group questions were first prepared in English, translated into Spanish, and then back-translated into English to ensure consistency. The final survey and focus group questions were pre-tested in a small group of CHWs. A final version of the survey and focus group questions was established following feedback from the pre-test. A bilingual, native Spanish speaker facilitator and two Spanish-speaking research assistants, including one of the two CHW that were members of the research team, conducted the focus groups. The online focus groups lasted ~60 min in duration and were conducted in the afternoons and/or weekends to accomomodate to the CHWs' work schedules. Three focus groups were conducted, including one in English and two that were facilitated in Spanish. Recent recommendations for conducting online focus groups over the COVID-19 pandemic suggest over-recruitment of participants to compensate for high attrition rates and technological difficulties (19); thus, focus groups in this project ranged in size between 14 and 15 participants. To ensure that all participants had a voice, we used a chat feature of the online platform to capture participants' comments. No compensation was provided for participation. The focus groups were audiotaped and participants consented to the audiotaping prior to participation. All audiotapes were transcribed by a professional service for analyses. The Spanish focus groups were first translated by the professional service, and subsequently transcribed. [Blinded] approved this project and provided exemption given that the project was deemed to be a community needs assessment (HCS20200306N).

### Measures

Consistent with community-engaged research, the survey questions were developed in collaboration with community partners from local CHW associations. Both, structured and open-ended questions were used. The survey included questions to assess: (a) demographic information (i.e., gender, age, race, ethnicity, and education); (b) working expertise and history as CHW (i.e., work status, work setting, credential/licensing information, years working as CHW, demographic characteristics of their clients/patients, types of communities where their work takes place, and financial support for their work); (c) health areas addressed in the CHW's work by indicating *yes/no* to a comprehensive list of physical and mental health areas including chronic medical conditions, lifestyle/health behaviors, mental health and prevention health; (d) perceived barriers to the CHW's work by indicating *yes/no* to a comprehensive list of barriers documented in the literature as limiting access to healthcare among historically underserved and low-income communities (20); and (e) perceived mental health training needs to facilitate the work of CHWs, which was done by indicating *yes/no* to a list of mental health training areas in line with Guidelines recommended by the American Psychological Association for working with people with low-income and economic marginalization (21). Open-ended questions were used to assess for any domains that may have not been included in the lists of barriers and mental health training needs to the work of CHWs.

For the qualitative data, a topic guide was developed in collaboration with local CHW associations, which was used to guide the discussions for the focus groups. Questions for the focus groups were semi-structured and were aimed at fostering discussion pertaining to the: (a) experience of distress from the COVID-19 pandemic and relevant mental health concerns; (b) identification of barriers faced by CHWs in the provision of mental health services to the community; and (c) identification of training needs for CHWs to more effectively provide mental health resources, referrals, and recommendations for historically underserved communities. As recommended by our collaborating CHWs, at the end of each interview or group, we provided participants with an opportunity to provide us with anonymous feedback using a comments and suggestion sheet. This was extremely helpful and provided us with knowledge that we incorporated into subsequent groups (e.g., provide information about resources for local mental health and legal services; explain the importance of research; schedule future talks in the community; longer duration of the groups to allow for more in-depth discussion).

### Analyses

Quantitative data were analyzed using SPSS software. Descriptive statistics were used to develop a demographic profile of participating CHWs and the communities they serve. Frequencies and percentages were used to identify barriers and training needs to the work of CHWs. Over a two-month period, weekly group meetings with the principal investigator, research assistants, and collaborating CHWs were scheduled to analyze the qualitative findings. Qualitative data was coded by four members of the research team including the principal investigator, one research assistant, and the two CHWs members of the research team. Qualitative data from the focus groups were analyzed through systematic methods as outlined by Miles and Huberman (1994) by starting with specific questions previously developed and then proceed through data categorization, data reduction, data display, and conclusion drawing and confirmation using triangulation (22). More specifically, we began by using a socio-ecologic approach as template to categorized barriers faced by CHWs into themes, specifically (a) personal barriers, (b) community barriers, (c) environmental barriers, and (d) organizational barriers. To reduce the data into primary codes and subcodes, we used a table displaying primary themes. To confirm the categorization and validity of the definitions given for each of theme, primary code and subcode, we engaged in data triangulation by comparing survey data with data, notes and observations from the focus groups (22). Also, upon completion of data analyses, the results described below were presented and discussed with our collaborating CHW organizations, and modifications were made with the feedback provided.

## Results

### Participants

Participants were 43 CHWs from a region near the U.S.-Mexico border (see Table 1).

**Table 1:** Characteristics of CHWs.

**Characteristics**	**Participants (*N* = 43) (*M, SD*) or %**
Age (*M, SD*)	45 (11.1)
**Sex**	
Women	93%
**Ethnicity**	
Latinx	91%
**Education**	
>High school	88%
**Employment**	
Full or part time	81%
Length as CHW (*M, SD*)	7 (6.8)
Certified CHW	91%

M, Mean; SD, Standard Deviation.

Most were women (93%) and of Latino origin (91%). The average age of participating CHWs was 45 years (SD = 11.1). Most CHWs had graduated from high school and were working full or part-time as CHWs. On average, participants had been working as CHWs for 7 years (SD = 6.8) and the majority were certified. In Texas, certification for CHWs requires training in nice core competencies including communication skills, interpersonal skills, service coordination skills, capacity building, advocacy skills, teaching skills, organizational skills and basic knowledge on specific health issues with a primary focus on physical health (23).

Most participants reported working with low-income communities that face significant barriers accessing healthcare (81%), the majority worked in rural and/or remote settings (63%), and approximately half reported working with immigrant communities, including working with families with undocumented immigration legal status (49%). Most reported working across various settings including community locations (67%), non-profit offices/facilities (51%), churches or faith-based centers (33%), and primary care clinics (26%). Regarding the health areas that these CHWs address in their communities, the majority reported that their work addressed mental health and wellness needs of members in their community (*n* = 31.72%) (see Table 1).

### Barriers faced by CHWs in assisting community members with their mental health needs

Results from the quantitative data showed that 56% of CHWs reported inadequate and insufficient mental health resources as a salient barrier that interferes with their ability to assist community members with their mental health needs, followed by cultural factors (49%), undocumented immigration legal status (47%), fear (42%), mistrust (35%), and limited opportunities to receive mental health training (30%). These findings are consistent with qualitative data gathered from the focus groups, which identified barriers at four levels of influence: personal, community, environmental, and organizational (see Table 2).

**Table 2:** Barriers to the work of CHWs, their consequences, and sample quotations.

**Level**	**Barriers**	**Consequences**	**Sample quotations**
Personal	Competing demands • Family obligations • Personal losses • Financial difficulties	Distress/burnout Diminished work capacity	We're trying to help [people] but we need to make sure we're careful with ourselves also… we can't really give all of our energy out because when we do then we're going to run out ourselves, and then instead of trying to help, we might end up being patients as well.
	Fear • Contagion and spreading of COVID-19	Distress	Going around other people gives us concern [about] getting exposed… it's feeling the guilt of knowing that I can be responsible if somebody else gets sick.
	Workload • Large • Constant	Distress/burnout	There is always the fear of leaving people halfway… it is very difficult because a person may say, “she never answered me” or “I asked her for help and she was never there for me”… all I have is my word.
	Overwhelming responsibility	Distress/burnout	[People] tell me that I'm the only constant in their lives right now since the pandemic.
Community	Fear/Mistrust among community members • Confusing information • Misuse of information	Difficult to build trust Confusion Distress	A lot of my clients don't know what to believe… they don't know who to believe or what is a good source of information… everybody says something different… there is a lot of confusion and [people] look to us for the answers, but it is difficult.
	Cultural factors • Limited English proficiency	Isolation Difficulty providing referrals and resources	“We are very uncertain because we do not know what to do… they know absolutely no English.”
	Exacerbation of pre-existing stressors • Physical illnesses	Distress Delay in accessing services	“The body is reacting to what the mind cannot longer [process] due to so much thinking… It is like a snowball that grows, unfortunately.”
	Mental health stigma	Isolation Fear Difficulty providing referrals and resources	With what is happening today with the pandemic, there is much more stigma surrounding mental health in the community… in our society, mental health is looked at as something bad… as if there's something wrong with you… because of that stigma, people don't reach out to us.
Environmental	Limited ability to pay for mental health services	Distress Financial burden Difficulty providing referrals and resources	Down here where we are, it's very poor and there's a lot of sickness and sorrows and struggles with physical health and with mental health.
	Undocumented immigration legal status	Distress Mistrust Isolation Difficulty providing referrals and resources	People fear seeking help because of their immigration status… immigration is a big factor… many people don't want to give their information in the agencies databases.
	Technology • Limited or lack of access • Difficult to use • Generational barriers • Sharing of limited resources	Isolation/marginalization Difficulty providing referrals and resources	Many people do not have access to technology… also, clients are often overwhelmed with the phone… they don't know how to check their voice messages and they don't know how to open documents… many times they have their phone, but they don't know how to use it.
Organizational	Limited mental health resources and/or services • Scarcity • Not culturally tailor • Not addressing contextual issues	Distress Burnout among CHWs Difficulty providing referrals and resources	People are getting depressed and anxious… sometimes I feel helpless that I can't give them the help I want to get them… we need resources that have more information.
	Limited funding • Scarce • Short-term	Burnout among CHWs Disruption in the continuity of services Mistrust	When a program is over, it affects [CHWs] because people continue to trust us… we have to keep following up with them and provide references, and then we don't know where to go… the program closes, but we do not stop working… for us as CHWs it is very difficult.
	Limited training	Burnout among CHWs Distress Isolation	We need training on how to deal with difficult situations because sometimes one wants to do more, but we cannot because we do not have enough tools or training.

At the personal level, CHWs identified four barriers interfering with their ability to help including, (a) competing personal demands, such as family obligations, personal losses, and financial difficulties; (b) fear of contagion and spreading of COVID-19 to loved ones and to the community; (c) constant and large workload due to excessive community needs; and (d) overwhelming sense of responsibility toward the community. These personal barriers increase distress and burnout among CHWs.

At the community level, CHWs identified four barriers interfering with their work. These included, (a) mistrust from the community as a result of confusing information and concerns about misuse of information and violation of confidentiality; (b) cultural factors, particularly limited English proficiency; (c) mental health stigma that is prevalent in the community, and (d) exacerbation of pre-existing stressors such as physical illnesses that lead community members to prioritize physical over mental health needs. Overall, the aforesaid barriers increase distress and make CHWs job more difficult as they work with complex cases while trying to overcome their client's mistrust, fears, skepticism, isolation, limited ability to access resources, and low willingness to seek mental health treatment.

Three environmental barriers were identified, including, (a) limited ability to pay for mental health services, which interferes with the referral process and increases financial burden; (b) immigration legal status, given that a considerable proportion of the community is undocumented or have family members or friends who are undocumented, which leads to fear of disclosing information and increases avoidance toward seeking services; and (c) limited access to technology, including having to share phones, computers or software, having difficulty using technological equipment or navigating online sites, and bridging the generational divide. The technological barriers can lead to increased isolation and marginalization among vulnerable community members (e.g., elderly, impoverished, and undocumented immigrants), as well as create difficulties in accessing health information, resources and services.

Barriers at the organizational level were identified, including, (a) limited referral sources and inadequate resources due to scarcity and a lack of culturally and contextually appropriate resources; (b) limited funding to support CHWs' work, including funding that is short-term and interrupted; and (c) limited training opportunities to equip CHWs with skills to address the mental health needs of their clients. Overall, organizational barriers contribute to increase burnout among CHWs and limit their work capacity and abilities.

### Training needs of CHWs to address mental health in historically underserved communities

**Figure 1:**
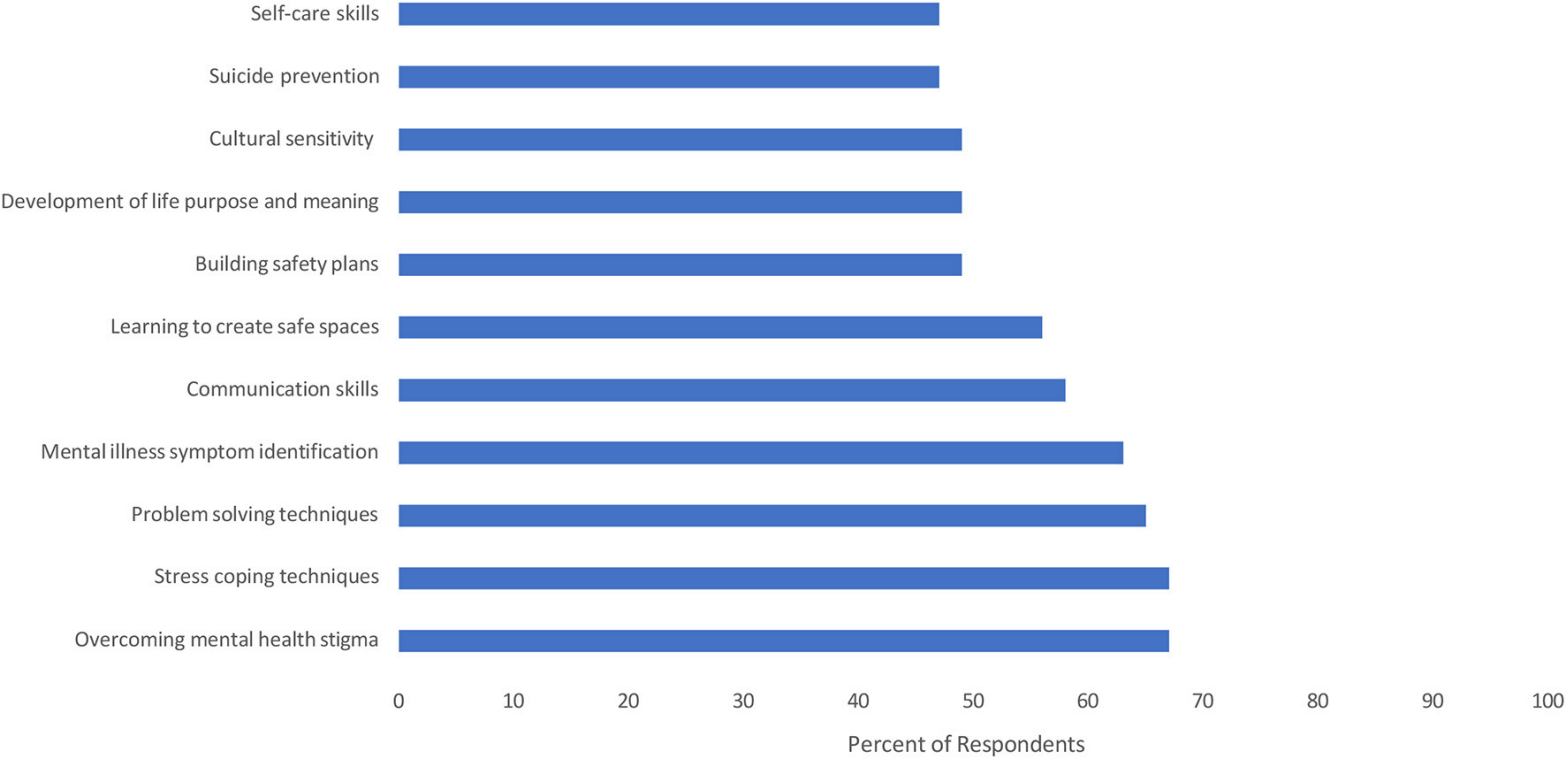
Training recommendations for CHWs to address mental health needs in underserved communities.

Results from the quantitative data showed that on average, CHWs reported that addressing mental health in their communities is of primary importance (*M* = 9.5 out of 10, *SD* = 1.3). The quantitative survey showed that top five training needs pertained to skills aimed at overcoming mental health stigma (as indicated by 67% of participants), teaching of stress coping techniques (67%), learning problem solving techniques (65%), symptoms identification (63%), and communication skills (58%) (see Figure 1). The qualitative data supported the aforesaid results while also providing insights into additional training needs. Overall, nine needed core training areas were identified through the focus group discussions (see Table 3).

**Table 3:** Themes highlighting CHWs' mental health training needs and sample quotations.

**Training need theme**	**Sample quotations**
Communication	“*It's important for us to learn how to talk about difficult things*… *we need to listen to [people] more than we speak, and this does them a lot of good*… *I have realized that many times all people want is to let off steam*… *so I let them talk until they have a question and then I ask them what they would like me to do for them*… *it makes it easier for them to express themselves and I can more or less realize what their greatest needs are.” “To learn what we can say when we bring ourselves to these patients to comfort and console them, educate them during this time. However, with some [those who are undocumented] we need to work more closely because we have to earn their trust*… *knowing how to effectively gain this trust would be helpful*… *knowing what words will validate their emotions and [reduce] anxiety effects will be helpful.”* “*Listening*… *don't want to assume you know something … lending an ear*… *you might not always solve the problem but listening still helps.”*
Symptom identification/screening	“*[People] are getting sick*… *they feel desperate, they don't eat and then they want to commit suicide. We need to help them. “[CHWs] need more education on [mental] illnesses*… *tools*… *understanding because it's hard to help those with mental illness if you don't have proper education*… *it is something that can be worked with, but requires teamwork.”*
Trauma	“*We need to learn more about trauma and how it affects [ people]* … *I come from a family in which we weren't supposed to talk about certain things*… *our people need to learn that it's ok, that we can talk about these things, about things that have happened like trauma and abuse.” “Family violence is present more now with increased contact*… *[CHWs] feel helpless to help patients who are trapped in violent homes.” “[CHW] offers resources to these ladies, but they don't always know they were even being abused by their partners*… *sometimes, they tell [CHW] that since [CHW] was the one to open their eyes to the abuse, then [CHW] should be the one to give them resources to deal with this issue*… *the men, in turn, tell [CHW] that all [CHW] did was make his wife more combative, because she asks him to help him cook more often*… *it would be good to find a partnership with some clinic or organization that can offer resources like counselors or psychologists without asking for documentation, which is what most of the community members are afraid of”*
Stress reduction strategies	“*I think giving [our] communities the knowledge they need so that they can help themselves is really great*… *physical and mental health are connected and maintaining one benefits the other*… *exercise, sleep well, maintain good consistent routines to optimize mental wellness … these skills are very good to learn not only for [CHWs], but also to teach [our] clients.” “Tutorials on how to calm oneself and feel a sense of oneness” “Breathwork is one of the easiest things to teach people to destress, make clear decisions, etc*… *teaching breathwork for [CHWs] and to teach their clients*… *Mind-body connection is important.”*
Strength-based approaches	“*When you remember that child you have inside, you understand where many of your problems and strengths come from*… *to know why we are in the situation we are in today and how we can move forward is a good thing*… *this is very good to know if you're trying to draw someone to acknowledge and own what it is that might need to be changed.”*
Overcoming mental health stigma	“*We need to learn more, educate ourselves more about mental illness and mental health all together. That's not something that's covered in depth in becoming a CHW*… *it would be very important to know the difference between mental health and mental illness and knowing how to work with patients that are struggling and don't understand it.” “Patients need more information than is being given to the general citizenry, in Spanish-preferably*… *they also need medication referral information.” [Learn] not just the mental illnesses but also the history*… *learn what was done with mentally ill people over the times because this develops a sense of empathy in people.”*
Cultural sensitivity	“*I work with clients that have [indigenous] background, so that's something that I would like to learn a little bit more regarding their beliefs*… *their beliefs about grieving are very different from ours so that's something that I would benefit from*… *we need skills to help understand different cultural beliefs and views of mental illness and how [people] reacted in the past or how [people] deal with it now are needed so that we can better help them.”*
Information processing	“*For education, [people] do not know how to use Zoom, unlike the use of Facebook so that is the tool that I started to use for presentations.” “Facebook and zoom platforms would work and I've seen success with them; however, there is a learning curve to understanding how to use this tool, like others*… *my recommendation for the Colonias is to use Facebook because it is a more familiar platform*… *zoom isn't as good because [CHWs] would have to teach people how to use it*… *[CHWs] could utilize short, open platforms where people can comment and participate in the educational process*… *Facebook live events would be ideal for this process, ultimately.” “[People need] a phone number to call for assistance and referrals or on call-guidance from promotoras*… *The [phone] numbers previously given are not for the distributing organization that [people] trust.”*
Self-care	“*It is important to support [CHWs] once in a while to ignite us and encourage us for the work that supports our communities.” “[CHWs] need guidance on how to leave the community concerns “outside of the home” to protect [the] home environment.” “Making calls between people you already know is important, even just as a courtesy to check in on them*… *this is a type of therapy itself because it allows people to unburden themselves.”*

#### Communication

The relevance of learning communication skills was emphasized at two levels: (a) to facilitate communication between CHWs and their clients; and (b) to help clients develop communication skills to more effectively relate and interact with their loved ones and others in their community. The need to build skills to facilitate discussion of difficult topics was highlighted, specifically pertaining to issues related to mental illness, sexual health, domestic violence, undocumented immigration legal status, and discrimination/racism. To achieve this, participants emphasized the importance of learning active listening and skills to validate emotions.

#### Symptom identification and screening

Participants identified a need for building knowledge regarding symptom presentation and identification of common mental disorders including anxiety, depression, somatization, trauma, and stress-related disorders. This included learning how to administer easy-to-use screening tools to determine severity of the presenting problems and to facilitate referrals when needed. Participants also emphasized the need to learn about effective treatments for the aforementioned disorders in order to educate their clients about their alternatives and to be more effective when making referrals. Another important area that emerged regarding symptom identification and screening was suicide prevention, including the identification of risk factors, warning signs, and knowing when and where to make a referral.

#### Trauma

Given the high prevalence of trauma in their communities, participants highlighted the need to build knowledge about how trauma and stress-related disorders develop, how they are managed, and how to help people cope with trauma. The importance of learning about the short- and long-term consequences of trauma, as well as effective treatment options, were also brought up as essential knowledge that CHWs need to encourage their clients to seek treatment.

#### Stress reduction strategies

Learning different strategies that can be taught to clients to reduce stress was identified as fundamental. Given the high prevalence of somatization in their communities, CHWs emphasized the need to build knowledge helpful to understand the mind- body connection in response to stress, as well as specific stress reduction strategies, namely, (a) relaxation techniques (e.g., breathing exercises, meditation, visualization, imagery, mindfulness); (b) problem solving to facilitate decision making and reduce conflict; (c) building safety plans in cases of abuse, violence, health threats, environmental emergencies (e.g., floods and hurricanes) and for undocumented people living in fear of deportation; and (d) building support networks or healing circles within the community that can be self-sustaining sources of social support.

#### Strength-based approaches

Four important areas of resiliency-building skills were identified. First, CHWs identified the need to increase their skills in motivating clients to create and strive toward desirable goals despite facing adversity. In this regard, the value of techniques such as motivational interviewing was emphasized. Second, CHW s identified the need for skills to help people find purpose and meaning in life, particularly in the face of daring circumstances, such as the use of storytelling or life narrative techniques. Third, learning techniques to identify important values to facilitate coping with adversity and to motivate needed or desired changes was also highlighted, with religiosity or spirituality identified as a central value. CHWs commented on the need for skills that can help clients resolve ambivalence about their spiritual/religious concerns, as well as how to help clients keep strong in their faith without overstepping boundaries.

#### Overcoming mental health stigma

Participants identified the need for learning to distinguish between mental health and mental illness, and how to use this knowledge to reduce stigmatization and debunk existing stereotypes, myths and beliefs. CHWs also emphasized that building knowledge about the etiology of different mental illnesses and the factors that increase risk, as well as information about the effectiveness of treatments would be helpful to overcome stigma and to normalize mental illnesses. Given the high stigma surrounding psychiatric medication in their communities, participants also expressed a need for learning more about the role of medications in the treatment of mental illnesses, particularly when integrated with complementary and alternative approaches that may be more acceptable to the community (e.g., supplements, herbal remedies, and body exercises).

#### Cultural sensitivity

Four primary training areas emerged in this regard. First, a need to learn how cultural factors impact mental health and treatment preferences, particularly when CHWs make referrals. CHWs need to learn about the role of alternative medicine or healing practices and how these may vary by culture. Second, participants expressed a need for learning about interpersonal dynamics and how these may vary across cultures. Two examples that emerged from the discussions were how gender may influence emotional expression and how cultural values may guide interactions among people. Third, CHWs reported a desire to learn techniques that can help discuss challenging myths and beliefs about mental health in a way that is respectful of a client's cultural background and experiences. Finally, relevant to the current pandemic and the increase in the number of deaths, CHWs mentioned a need to learn about different cultural views on death and dying, as well as bereavement rituals and practices in order to be sensitive to their clients' needs.

#### Information processing

Three areas related to information seeking, delivery and organization emerged. First, CHWs discussed the need to assist clients in learning how to seek reliable sources of information and how to evaluate the quality of such information. Second, CHWs identified a need to teach clients how to use social media effectively and safely given that many of the community members use this as a primary channel for information. Next is the need for developing skills or strategies to build a resource library.

#### Self-care

Three primary training areas emerged regarding self-care. First, training in symptom identification and management of burnout was noted as essential, as well as strategies for preventing burnout and for building self-care into everyday life. Another relevant area was the development of skills to build assertiveness and healthy boundaries, such as learning to say “no” and learning to achieve work-life balance. Finally, many participants identified the need to build support systems so CHWs can be in close contact with other CHWs to discuss personal matters and consultation on complex cases.

Table 4 provides an outline of recommendations for specific skills related to mental health that may address the aforesaid training needs of CHWs.

**Table 4:** Recommendations for CHWs' mental health training needs.

**Recommended domains of training needs**	**Skills**	**Description**
Communication	Discussion of sensitive topics	Build skills to facilitate the discussion of sensitive topics: promoting dialogue; building safe spaces; fostering resilience; problem solving
	Active listening	Build skills to become a good listener: paraphrasing skills; echoing skills; probing techniques; reading cues
	Validation	Develop skills helpful to build trust and convey understanding, support and acceptance of a person's experiences or situations: demonstrating empathy; validating emotions; offering encouragement if appropriate; avoiding invalidation
Symptom identification/screening	Anxiety; depression; somatization; trauma and stress related disorders	Build knowledge about symptom presentation; identification and use of practical screening tools to determine severity; learn about effective treatments to facilitate referrals; identify appropriate referral sources
	Suicide prevention	Build knowledge about risk factors, warning signs, making referrals as needed
Trauma	Effects of trauma and abuse	Build knowledge of how trauma related disorders develop and are maintained; risk factors; short and long- term consequences of trauma and abuse
	Management	Build knowledge about treatment; resilience building strategies; prevention of re-traumatization
	Safety planning	Learn the components, steps, and implementation of safety plans, including how to ask for help in cases of abuse, violence, health threats, environmental emergencies, and immigration-related concerns (e.g., deportation)
Stress reduction strategies	Mind-body connection	Lean about the mind-body connection in response to stress
	Relaxation techniques	Learn and master relaxation strategies: breathing exercises (e.g., deep breathing and abdominal breathing); progressive muscle relaxation; use of imagery and visualization; meditation; mindfulness
	Problem solving	Learn skills to problem solve: definition of a problem; generation of solutions; choosing a solution; implementing the chosen solution; evaluating outcomes; reviewing the process; steps to conflict resolution
Strength-based approaches	Increase motivation	Learn to use motivational interviewing to facilitate desirable life changes
	Find purpose and meaning in life	Learn to use techniques such as life narrative to facilitate the development of identity, purpose and life meaning
	Value-based living	Learn to identify values for coping with adversity and to foster resilience; use values as sources of motivation toward desirable life changes
	Religiosity and spirituality	Develop skills to remain strong in the faith; assisting with spiritual ambivalence
Overcoming mental health stigma	Mental health	Learn the difference between mental health and mental illness; mental health promotion as prevention of mental illness; factors that contribute to stigmatization; negative effects of stigmatization demystifying maladaptive myths/beliefs
	Etiology of mental illnesses	Learn about risk factors contribute to mental illnesses; protective factors; normalizing mental illnesses
	Treatment of mental illnesses	Build knowledge about the effectiveness of mental health treatments; demystifying the use of psychiatric medication; validating the role of complementary and alternative medicine; effective use of testimonials to overcome stigma
Cultural sensitivity	Acknowledge cultural variations	Learn how cultural factors impact mental health and treatment seeking behaviors; consider cultural factors in making referrals; assessing and addressing the role of alternative medicine and/or healing practices
	Interpersonal dynamics (i.e., gender roles)	Learn to relate effectively with people of different backgrounds; learn to build trust and promote shared understanding; assist in bridging the cultural gap when navigating health services
	Addressing myths and beliefs	Learn to use open-ended questions and probing techniques to explore unique outlooks and relevant myths/beliefs about mental health and mental health treatment
	Bereavement	Learning about different views on death and dying; loss and grief; culturally appropriate practices and rituals; bereavement preferences; make appropriate referrals
Information processing	Information seeking	Learn to identify reliable sources of information; learn to evaluate the quality of information sources
	Information delivery	Learn to safely and effectively use social media; identify alternative sources of information delivery to match community needs
	Information organization	Identify skills and strategies to build a resource library
Self-care	Burnout	Learn to identify and manage symptoms; prevention strategies; stress management; benefits of self-care; barriers to self-care
	Assertiveness training	Build skills to develop healthy boundaries; work-life balance; learn to say “no”
	Building support	Identify and/or create support systems, including consultation services

## Discussion

This project highlights barriers that inhibit the ability of CHWs to effectively address the mental health needs of historically underserved communities near the U.S.-Mexico border region. The barriers identified, which exist on the personal, community, environmental and organizational levels, range from competing personal mental health needs and obligations to excessive workload and lack of resources/funding. At each level, participants described the consequences for themselves and their clients. For CHWs, the consequences primarily revolved around increased distress from personal losses and experiences of hardship from the COVID-19, and excessive work demands due to prevalent mental health needs in their communities, which contributed to an increased sense of social isolation and burnout. For their clients, the consequences included decreased access to and utilization of vital services contributing to worsening of health outcomes, along with increased fear, distress, confusion, mistrust, feelings of stigmatization and discrimination, and social isolation. To address the barriers and consequences identified in this project, CHWs identified and described opportunities for training and skill building in core areas including communication, mental illness symptom identification, self-care and stress reduction, and cultural awareness and sensitivity.

Our findings bolster the evidence for acknowledging that CHWs are essential mental health workers in their communities who share similar experiences to the clients that they serve. CHWs are tasked with promoting the overall health and wellbeing of the people they serve, which by definition includes supporting not only physical, but also mental health (13). Despite their critical role in caring for their communities, our results indicate that CHWs often feel overwhelmed and that they do not feel adequately prepared to effectively address mental health needs in their communities despite certification requirements. Mental health training is needed for their self-care and to better care for their community members with psychological concerns. Indeed, CHWs may better assess their own mental health needs and those of their clients with appropriate training and support. Evidence-based trainings, including promotion of self-care, strengths-based approaches, cultural sensitivity training, and interviewing skills are key element of certification and training that need to be expanded so that CHW can more effectively work with people with complex needs and compounded stressors (13, 24, 25). Given the multifaceted nature of their roles, CHWs must be equipped to identify symptoms, manage stress levels and seek treatment when necessary—for both their community members and themselves.

As trusted members of the communities in which they work, CHWs may feel obligated to maintain relationships with clients long after funding and resources for programs have ended. These obligations can bear a heavy emotional toll as CHWs often share many of the same struggles faced by the communities where they work, which can be a significant source of stress and burnout. Therefore, it is essential that the mental health needs of CHWs are prioritized. Examples of effective mental health aid for CHWs can include stress management workshops, instruction on the establishment of health boundaries, teaching methods for self-care, and education to identify symptoms of stress and burnout (26).

To address barriers contributing to poor mental health amongst both CHWs and their clients, we recommend the following. First, the creation of supportive peer networks is essential to assist CHWs with professional consultation, personal development, and emotional support. Second, to address the growing needs of and health disparities facing historically underserved communities, we recommend significantly increasing the size of a CHW workforce that is supported in sustainable funded positions. We encourage funding agencies and health systems to develop more flexible and innovative funding streams that will allow CHWs to engage longitudinally, regardless of health topic or priority, with the communities they serve. Third, our health system must continue innovating and developing integrated health services programs where our clients and communities mental and social wellbeing is given equal priority and focus as to their physical health. CHWs are well-placed to help integrate and coordinate such services. Finally, to address mistrust and confusion, we recommend the utilization of multi-modal community outreach and education strategies, of which CHWs are just one component. Strategies must include content that is tailored to the specific culture and context of each community, and should include inter-personal communication through CHWs, other outreach workers, healthcare providers, and other trusted community voices, but also social medial, traditional media, print materials and other formats. Our strategies must also include training on how historically underserved communities choose information sources and validate the information they find.

This project has limitations. Our sample was a modest size, yet it was drawn from a large region comprising many underserved communities. Additionally, the surveys and focus groups were conducted over a videoconferencing platform, which may have excluded some participants. However, we gathered diverse perspectives from a broad region, in a manner that allowed for participation while following physical distancing during the pandemic. Despite these limitations, our results shed light on ways to support the work of CHWs and their important contribution to addressing growing mental health concerns among vulnerable communities.

Future research should examine which training methods are best for enhancing mental- health learning for CHWs. For example, developing a better understanding of psychological assessments and resources for CHWs and their clients is of primary importance. Additional studies should examine effectiveness of training in terms of CHW skill-building and client outcomes, as well as identifying avenues for the sustainability of programs led by CHWs (27). This includes avenues for the provision of tele mentoring training and services to rural and remote areas. Importantly, research on preventing and addressing burnout in this population is also lacking and yet, needed.

In conclusion, CHWs are an essential part of the healthcare workforce. Addressing mental health should be an integral part of the COVID-19 response among historically underserved communities, which have been disproportionately affected. CHWs are facing new challenges with limited resources in order to assist the complex mental health needs of those they serve. The tasks performed by CHWs require a unique skill set and a significant emotional investment. Mental health training is required for CHWs to effectively carry out their work and maintain their own wellbeing. It is time to invest in the mental health training and support for these essential workers to ultimately further health equity and to protect the mental health of the most vulnerable during and beyond the COVID-19 pandemic.

## Data availability statement

The raw data supporting the conclusions of this article will be made available by the authors, without undue reservation.

## Ethics statement

The studies involving human participants were reviewed and approved by the University of Texas Health Science Center at San Antonio Institutional Review Board. The patients/participants provided their written informed consent to participate in this study and to the audiotaping of the focus groups prior to participation.

## Funding

Funding was provided by Grants from the National Heart, Lung, and Blood Institute (NHLBI) of the National Institutes of Health (NIH) (K01HL150247; PI: LG) and the National Institute of Diabetes and Digestive and Kidney Diseases (NIDDK) of NIH (K23DK123398; PI: KK).

## Conflict of interest

The authors declare that the research was conducted in the absence of any commercial or financial relationships that could be construed as a potential conflict of interest.

## Publisher's note

All claims expressed in this article are solely those of the authors and do not necessarily represent those of their affiliated organizations, or those of the publisher, the editors and the reviewers. Any product that may be evaluated in this article, or claim that may be made by its manufacturer, is not guaranteed or endorsed by the publisher.

## Author disclaimer

The content is the responsibility of the authors and does not represent the views of the NIH.
